# Follow-up of folinic acid supplementation for patients with cerebral folate deficiency and Kearns-Sayre syndrome

**DOI:** 10.1186/s13023-014-0217-2

**Published:** 2014-12-24

**Authors:** Pilar Quijada-Fraile, Mar O’Callaghan, Elena Martín-Hernández, Raquel Montero, Àngels Garcia-Cazorla, Ana Martínez de Aragón, Jordi Muchart, Ignacio Málaga, Rafael Pardo, Pedro García-Gonzalez, Cristina Jou, Julio Montoya, Sonia Emperador, Eduardo Ruiz-Pesini, Joaquín Arenas, Miguel Angel Martin, Aida Ormazabal, Mercè Pineda, María T García-Silva, Rafael Artuch

**Affiliations:** Unidad de Enfermedades Mitocondriales-Enfermedades Metabólicas Hereditarias. Dpto. de Pediatría y Radiología, Hospital 12 de Octubre, Madrid, Spain; Centre For research in rare diseases (CIBERER), Institut de Salud Carlos III, Madrid, Spain; Pediatric Neurology, Clinical Biochemistry, Histopathology and Radiology Departments, Hospital Sant Joan de Déu, Passeig Sant Joan de Déu, 2., Esplugues, Barcelona, 08950 Spain; Servicio de Pediatría, Hospital Universitario Central de Asturias, Oviedo, Spain; Servicios de Pediatría y Radiología, Hospital de Cabueñes, Asturias, Spain; Departamento de Bioquímica, Biología Molecular y Celular, Universidad de Zaragoza, Instituto Aragonés de Ciencias de la Salud, Zaragoza, Spain; Mitochondrial Diseases Laboratory, Hospital 12 de Octubre Research Institute (i + 12), Madrid, Spain

**Keywords:** Kearns-Sayre syndrome, Mitochondrial DNA deletion, Cerebral folate deficiency, Folinic acid treatment, Neuroimaging

## Abstract

**Background:**

Kearns-Sayre syndrome (KSS) is a mitochondrial DNA deletion syndrome that presents with profound cerebral folate deficiency and other features. Preliminary data support the notion that folinic acid therapy might be useful in the treatment of KSS patients. Our aim was to assess the clinical and neuroimaging outcomes of KSS patients receiving folinic acid therapy.

**Methods:**

*Patients*: We recruited eight patients with diagnoses of KSS. Four cases were treated at 12 de Octubre Hospital, and the other two cases were treated at Sant Joan de Déu Hospital. Two patients refused to participate in the treatment protocol.

*Methods*: Clinical, biochemical and neuroimaging data (magnetic resonance imaging or computed tomography scan) were collected in baseline conditions and at different time points after the initiation of therapy. Cerebrospinal fluid 5-methyltetrahydrofolate levels were analysed with HPLC and fluorescence detection. Large-scale mitochondrial DNA deletions were analysed by Southern blot.

*Treatment protocol*: The follow-up periods ranged from one to eight years. Cases 1–4 received oral folinic acid at a dose of 1 mg/kg/day, and cases 6 and 8 received 3 mg/kg/day.

**Results:**

No adverse effects of folinic acid treatment were observed. Cerebral 5-methyltetrahydrofolate deficiencies were observed in all cases in the baseline conditions. Moreover, all three patients who accepted lumbar puncture after folinic acid therapy exhibited complete recoveries of their decreased basal cerebrospinal fluid 5-methyltetrahydrofolate levels to normal values. Two cases neurologically improved after folinic therapy. Disease worsened in the other patients.

Post-treatment neuroimaging was performed for the 6 cases that received folinic acid therapy. One patient exhibited improvements in white matter abnormalities. The remaining patients displayed progressions in subcortical cerebral white matter, the cerebellum and cerebral atrophy.

**Conclusions:**

Four patients exhibited clinical and radiological progression of the disease following folinic acid treatment. Only one patient who was treated in an early stage of the disease exhibited both neurological and radiological improvements following elevated doses of folinic acid, and an additional patient experienced neurological improvement. Early treatment with high-dose folinic acid therapy seems to be advisable for the treatment of KSS.

**Trial registration:**

EudracT2007-00-6748-23

**Electronic supplementary material:**

The online version of this article (doi:10.1186/s13023-014-0217-2) contains supplementary material, which is available to authorized users.

## Background

Mitochondrial DNA (mtDNA) single-large deletion syndromes predominantly comprise three phenotypes: Kearns-Sayre syndrome (KSS: ORPHA480), Pearson syndrome (ORPHA699), and different subtypes of progressive external ophthalmoplegia [1]. Kearns-Sayre syndrome is a multisystemic disorder caused mainly by single deletions in mtDNA [2]. The clinical features of these syndromes have been extensively studied over the last decades. Although mtDNA deletions are present in different tissues of KSS patients, the central nervous system (CNS) is the most severely affected system.

The choroid plexus is a target organ of KSS. The choroid plexus is the main site of active folate transport to the CNS [3]. In addition to this function, the choroid plexus has other roles in the active transport of essential micronutrients from the blood into the cerebrospinal fluid (CSF) and also transports many exogenous chemicals and waste products from brain metabolism out of the CSF [4]. Analyses of the CSF of KSS patients have shown high lactate and protein levels, dramatic reductions in 5-methyltetrahydrofolate (5-MTHF) in most cases, and other biochemical alterations [5,6]. It has been suggested that the accumulation of mutated mtDNA copies in the choroid plexus plays an important role in causing the increases in CSF protein levels and reductions in 5-MTHF levels that are characteristic of KSS [7]. The association between cerebral folate deficiency and KSS was first described in 1983 [8,9]. It was recently reported that cerebral folate deficiency is a common feature of KSS and other mitochondrial disorders [10,11]. Pineda et al. [5], described a positive clinical response following folinic therapy in a patient with a mtDNA deletion and cerebral folate deficiency. Preliminary data support the notion that folinic acid therapy may be useful for the treatment of KSS patients who present with cerebral folate deficiency, although no long-term follow-up studies have been reported. This statement is supported by the essential role of folate in brain metabolism because folate participates in hundreds of methylation reactions, and folate is essential for the methylation and stability of myelin [5].

The objective of this study was to assess the clinical and neuroimaging outcomes and biochemical data of patients with Kearns-Sayre syndrome who presented with cerebral folate deficiency and were treated with folinic acid.

## Methods

### Patients

Design: open label study. We recruited eight patients with diagnoses of mtDNA single large-scale deletion syndrome and cerebral folate deficiency. Case 1 had Pearson’s syndrome at onset but evolved to a complete form of KSS. All cases fulfilled the criteria for KSS during the time of the study. Data from some of the patients have been published elsewhere [5,11,12]. The patients were recruited at the Hospital 12 de Octubre (Madrid), Hospital San Joan de Déu (Barcelona) and Hospital Central de Asturias (Oviedo). Cases 1–4 were treated at 12 de Octubre Hospital, and cases 6 and 8 were treated at Sant Joan de Déu Hospital. Two cases (5 and 7) refused to participate in the treatment protocol but were included in the baseline patient descriptions (see the tables).

The ages, ages at disease onset, ages at the initiation of folinic acid supplementation, clinical assessment by the Newcastle Mitochondrial Disease Ratings Scale, CSF folate values, clinical features and the results of molecular studies (% mtDNA deletion and size) and histopathological studies of muscle biopsies of the patients are provided in Table 1.

**Table 1:** **Age, age at disease onset, age at the initiation of folinic acid treatment, CSF folate values, clinical features, molecular studies (% deletion and size) and histopathology on muscle biopsy**

	**Case 1** ^a^	**Case 2** ^a^	**Case 3** ^a^	**Case 4** ^a^	**Case 5**	**Case 6** ^a^	**Case 7**	**Case 8** ^a^
**Age (y)**	12	16	23	20	42	14	29	16
**Onset age (y)**	0.3	10	10	6	9	9	8	7
**Age of folinic acid treatment**	8.5	11	17	14	-	13	-	8
**CSF Folate (nmol/L)**	7	5.6	2	6	24	4	1	8
**Clinical features and age**								
**NPMDS Score** ^**b**^	31	7	26	29	100 (at 33y)**	17	114**	20
**Ophthalmoplegia and retinopathy**	7 y	10 y	10 y	7 y	18 y	9 y/12 y	10 y	9 y
**Cerebellar ataxia**	7 y		15 y	7 y	27 y	9 y	8 y	7 y
**Myopathy**	7 y	10 y	15 y	7 y	12 y	9 y	10 y	8 y
**Cardiac conduction block**	9 y	11 y	14 y	9 y	30 y	14 y	12 y	
**Diabetes mellitus**			12 y	12 y			15 y	
**Hearing loss**	+	12 y		14 y	21 y		11 y	9 y
**Others**	Renal Fanconi 2 y Exocrine pancreatic insufficiency 3 y Suprarenal insufficiency 7 y			Pacemaker	Myoclonic jerks Short stature 9 y	Short stature Renal insufficiency	Short stature Pacemaker at 15 y	Myoclonic jerks
								Partial seizures
								Short stature
								Renal insufficiency
								Suprarenal insufficiency
**Molecular studies: % deletion (size)**	75%	18%	40%	71%	77%	80%	62%	73%
	6900	7900	7100	5100	2434	5800	4977	4121
**Histopathology Muscle biopsy**	-	Normal	Lipids in fibers	Raged-red fibers (RRF), COX negative	RRF	-	RRF	RRF partial COX deficient-fibers

Not performed (−). ^a^Case that agreed to participate; ^b^At baseline (before folinic acid treatment). **Score assessed in adult patients using NMDAS.

### Methods

Clinical, biochemical and neuroimaging data (MRI or CT scan) were collected in baseline conditions and at different points after the initiation of therapy (Tables 1, 2 and 3). General and neurological physical examination was performed during visits that occurred at six-month intervals. Clinical outcome was assessed using the Newcastle Paediatric Mitochondrial Disease ratings Scale (NPDMS) in patients below to 18 years and the Newcastle Mitochondrial Disease Adult ratings scale (NMDAS) when patients reached adult age [13,14]. The NPDMS is a scale developed for and validated in children with inherited mitochondrial diseases to assess disease severity. Appropriate age-specific versions of the NPMDS were utilized: 2–11 years; and 12–18 years. Sections I-III which assesses organ-specific function was completed in each patient at baseline (before treatment) and during follow-up. Complementary exams (electroencephalograms, echocardiograms, electrocardiograms, visual and acoustic evoked potentials) and renal and endocrine examinations were performed depending on the clinical condition of the patients. The follow-up periods ranged from one to eight years.

**Table 2:** **Biochemical data and folinic acid therapy in KSS patients**

**Case**	**Age at the start of folinic acid treatment (years)**	**Folinic acid doses (mg/kg/day)**	**Time of therapy between lumbar puncture (years)**	**Plasma folate (ng/ml) NV: 2.6-18.7**	**Plasma lactate (mmol/L) NV:0.5-2.3**	**CSF values before and after therapy**		
						**5-MTHF (nmol/L) NV: 35-124**	**Lactate (mmol/L) NV: <2.8**	**Protein (mg/dl) NV: 15-41**
**1**	8.5	1	0.8	25	3	7➔48	5.1/-	115/-
**2**	11	1	3.7	-	1.5	5.6➔60	2.1➔1.4	121➔142
**3**	17	1	-	20	1.4	2	3.5	165
**4**	14	1	-	-	2.5	6	5.7	169
**5**	-	-	-	5.3	2.3	24	5.9	80
**6**	13	3	-	-	-	4	4.2	160
**7**	-	-	-	9.9	3.5	1	3.5	244
**8**	8	3	1	1.2	2.5	8➔82	5.2➔1.2	89➔117

CSF (cerebrospinal fluid), NV (normal values).

**Table 3:** **Neuroimaging (MRI or CT scan) data before and after the initiation of folinic acid therapy and clinical outcome**

**Cases**		**Time between images (years)**	**Atrophy**		**Abnormal high signal on T2-weighted images**					Clinical outcome NPMDS* Sections I-III Pre/Post
	**Age at neuroimaging before therapy**		**Cerebral**	**Cerebellar**	**Cerebrum subcortical**	**Brain stem**	**Basal ganglia**	**Thalamus**	**Cerebellum**	
			**Pre/Post**	**Pre/Post**	**Pre/Post**	**Pre/Post**	**Pre/Post**	**Pre/Post**	**Pre/Post**	
**1**	8	0.7			+ / ++	+/+	+/+		+/+	31/40
**2**	11	2.7			+/ ++	+/+		+/+		7/14
**3**	17	3	−/+		+	+/+	−/+	+	−/+	26/54**
4*	14	3	−/+		−/+		+/+		−/+	29/67**
**5**				+	+	+	+	+	+	Unknown
**6**	13	2				+/+	+/+		−/+	17/22
**7**			+				+	+	+	Unknown
**8**	8	2			+/ -					20/24

*CT scan, + (lesion), ++ (progression), − (not lesion or improvement). ** Score assessed in adult patients using NMDAS.

Biochemical methods: Plasma lactate and folate concentrations were measured with standard automated procedures. Lumbar punctures were performed to CSF in all cases according to a previously reported protocol [15]. CSF lactate and total protein concentrations were analysed with automated spectrophotometric procedures, and CSF 5-MTHF was determined by HPLC with fluorescence detection according to previously reported procedures [15].

#### Genetic analysis

DNA was isolated from the muscle in all cases with the exception of case 1 in which DNA was isolated from the blood. The mtDNA large-scale deletions were analysed by Southern blot.

Brain magnetic resonance imaging (MRIs) were performed in a 1.5 T Philips® or a 1.5 T General Electric® machine, and the protocol included T1- and T2-weighted images, diffusion weighted imaging (DWI), MR spectroscopy and fluid attenuated inversion recovery (FLAIR). MRIs were performed in all cases with the exception of case 4 who did not under MRI because of a pacemaker. A computed tomography (CT) scan was performed in this case. We evaluated the images before and at different times following folinic acid treatment (Table 3).

#### Treatment protocol

Cases 1–4 received oral folinic acid at a dose of 1 mg/kg/day, and cases 6 and 8 received higher doses (3 mg/kg/day, Table 2). The doses of folinic acid were selected according to previous reported protocols for the treatment of profound cerebral folate deficiencies [5,16,17]. Lumbar punctures following therapy were performed in three patients (cases 1, 2 and 8; Table 2). The remaining cases declined undergoing lumbar puncture.

Informed consent was obtained from the families of the patients. The ethics committees of the Sant Joan de Déu, 12 de Octubre and Central de Asturias Hospitals approved the study. The clinical trial was funded and approved by the Instituto de Salud Carlos III, Spanish Ministry of Health (EC07/90272).

## Results

No adverse effects were observed during folinic acid treatment. The clinical outcomes that followed are provided in Table 3. Two cases neurologically improved following folinic acid therapy (cases 6 and 8). In case 6, the NPMDS score worsened from 17 (at baseline) to 22 (2 years after folinic acid supplementation). This outcome was due to mild alterations in endocrine system (parathyroid hormone disturbances secondary to moderate renal function impairment) and asymptomatic changes in ECG (section II of NPMDS). Vision at night worsened secondary to the instauration of retinopathy. Interestingly, some neurological items (section III of NPMDS) remained stable, such as ptosis and eye movements, the mild myopathy with symmetrical weakness of hip and shoulder girdle. No neuropathy was developed. Remarkably, cerebellar ataxia and tremor improved. In case 8, the NPMDS score worsened from 20 (baseline) to 24 (2 years after folinic acid supplementation). This outcome was due to progression of hearing loss to severe deafness, secondary problems with communication, feeding problems with gastrostomy and the change of the educational achievement attending in a special school (section I of NPMDS). Mild constipation, asymptomatic ECG changes, mild renal function impairment and anemia were observed in this period (section II of NPMDS). Interestingly, some neurological items (section III of NPMDS) remained stable: mild myopathy with symmetrical weakness of hip and shoulder girdle unchanged and neuropathy remained moderate. A recuperation ad-integrum of visual conduction after visual evoked potentials study was surprisingly found, cerebellar ataxia and tremor improved, and the patient recovered ambulation. At the time of the initiation of treatment, this patient presented with an incomplete KSS syndrome and later fulfilled the criteria for this disorder (Table 1). The disease states worsened in the remaining cases. Case 1 died at age 13. Cases 5 and 7 were adults, refused to be treated and were lost during the follow-up period.

The biochemical data are displayed in Table 2. Severe cerebral 5-MTHF deficiencies were observed in all cases in the baseline conditions, with the exception of case 5, who presented with a moderate deficiency. The plasma folate levels were within the normal range except for cae 8. The CSF protein concentrations were greater than 100 mg/dL in six of the eight patients. Three patients agree to undergo lumbar puncture following folinic acid therapy (cases 1, 2 and 8), and the low CSF folate levels were reversed in all of these patients and reached normal values. The CSF protein concentrations remained elevated after treatment (Table 2).

**Figure 1:**
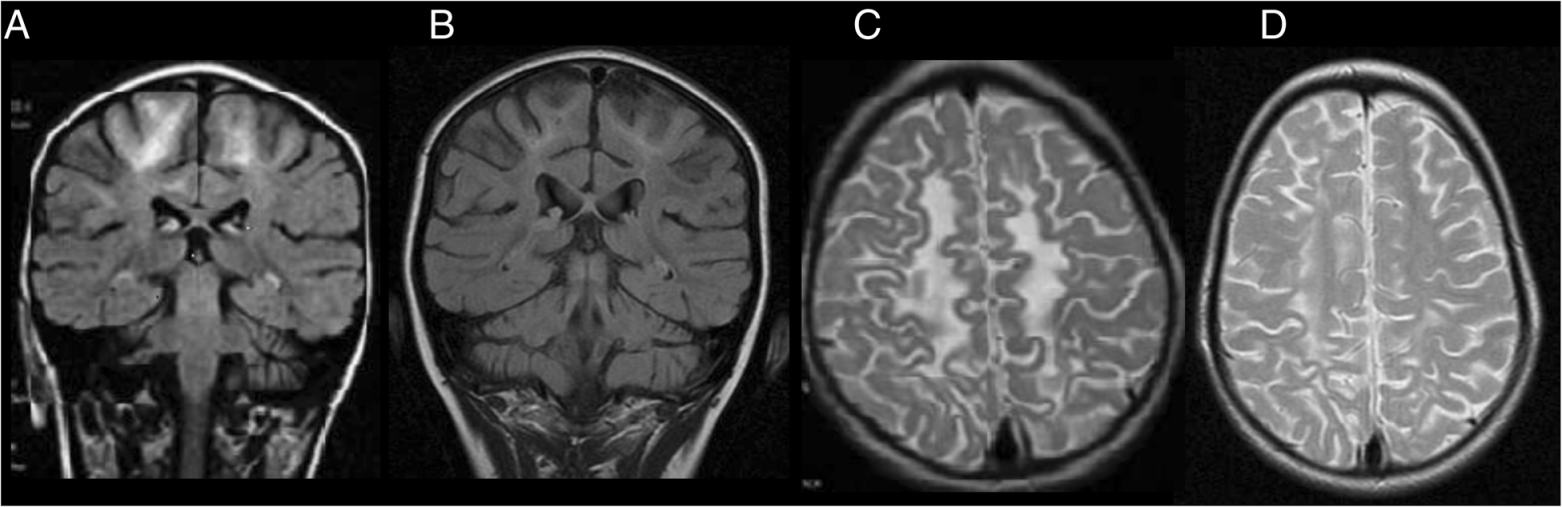
**(Case 8): MRI coronal T2W.** Abnormal high signal sparing the subcortical white matter with slightly periventricular location before folinic acid treatment were detected **(A)**, improving after 2 years of folinic acid treatment **(B)**. Abnormal high signal in the parietal white matter in MRI axial T2W was also observed before folinic acid treatment **(C)**. Decreased of high signal after 2 years of treatment with near normal white matter signal **(D)**.

Regarding the neuroimaging, at the time of the diagnoses of cerebral folate deficiency, the main features were abnormal MRIs with elevated signals in the T2-weighted images and the DWI images in the subcortical cerebral white matter, brain stem, basal ganglia (five out of eight cases) and thalamus (four out of eight cases). Cases 5 and 7 exhibited cerebellar and cerebral atrophy, respectively (Table 3). Neuroimaging following treatment was performed in the six cases who received folinic acid therapy. Case 8 exhibited improvement in the white matter abnormalities (Figure 1). The remaining patients exhibited progression in the abnormalities of the subcortical cerebral white matter, cerebellum and cerebral atrophy (Additional files 1, 2, 3, 4 and 5).

## Discussion

This is the follow-up of a series of KSS patients who were treated with folinic acid. This disorder is an example of choroid plexus failure in which the transport of folate from the blood into the CSF is inadequate and causes profound cerebral folate deficiency in the majority of cases [4,6,7]. Although the pathophysiology of KSS is complex and includes several aspects other than cerebral folate deficiency, the key role of this vitamin in brain function strongly supports folinic acid supplementation for these patients.

Clinical follow-up revealed that several neurological disturbances improved in cases 6 and 8, but the disease progressed in all patients evaluated by the Newcastle Scales, although very mildly in cases 6 and 8. One explanation for these findings is that brain is a target organ for folate deficiency in KSS patients, but probably not the other peripheral tissues. Moreover, the folinic treatment doses were higher for cases 6 and 8 (3 mg/kg/day). Although the post-treatment lumbar punctures that were performed in cases 1 and 2 (who received doses of 1 mg/kg/day) revealed that the 5–MTHF CSF levels reached normal values following folinic acid therapy, these values were still lower than that observed in case 8 (Table 2). It is difficult to exclude other factors potentially associated with the beneficial outcome observed in this work. Lactate values decreased in the second CSF sample in the 2 cases studied, but this observation may be anecdotic since it is not uncommon that lactate values are normal in mitochondrial disorders. Total protein values increased after folinic acid treatment, suggesting that the transport through choroid plexus remained impaired. Folinic doses from 0.5 to 1.8 mg/kg/day have previously been applied in certain non-mitochondrial diseases that present with cerebral folate deficiency and resulted in improvements in some neurological signs [16,17]. However, the underlying pathophysiological mechanisms of these clinical conditions might be different from those of KSS; consequently, the required folinic doses are presumably different.

Case 8 recovered ambulation and exhibited improvements in the MRI disturbances. This patient began treatment earlier than did the others with the exception of case 1, who began treatment at a similar age (8.5 years) but did not improve. However, case 1 presented with Pearson syndrome; therefore the phenotype of this patient was more severe than that of case 8. Furthermore, case 8 received folinic acid before he met the main clinical criteria for KSS syndrome. This patient was reported in 2006 [5] and exhibited abnormal subcortical white matter without basal ganglia or brain stem involvement on MRI at onset, which indicated that this patient was in the early stage of the disease. These findings suggest that this patient might have improved due to both the early treatment and the high dose of folinic acid. This finding indicates the importance of performing early lumbar punctures to detect cerebral folate deficiencies and initiate therapy as early as possible. Clinical improvements have been anecdotally reported in cases exhibiting cerebral folate deficiencies and mitochondrial disorders. A mitochondrial patient with mitochondrial respiratory chain complex I deficiency and cerebral 5-MTHF deficiency was treated with a folinic dose of 1.2 mg/kg/day. Hypotonia and ataxia improved in this patient, and the abnormal myelination patterns observed on neuroimaging were reversed following treatment for more than three years [18]. However, this patient exhibited a baseline CSF 5-MTHF value of 38.5 nmol/L, which is significantly higher than those observed in KSS.

The neuroimaging features observed in our cases are in accordance with those reported in other publications. Patients with KSS classically exhibit cerebral and cerebellar atrophy and high-signal lesions in the subcortical white matter on T2-weighted images [19] with or without symmetrical involvement of one or more areas that include the brain stem, globus pallidus, thalamus and cerebellum [20]. White matter involvement is predominantly peripheral and includes early involvement of the subcortical U fibres and sparing of the periventricular fibres [21]. The primary involvement of the subcortical white matter discriminates KSS from other white matter diseases such as lysosomal and peroxisomal disorders in which the subcortical regions are affected only later in the process [22]. In young adults or children, the combination of bilateral high-signal lesions in the globus pallidus and high-signal foci in the subcortical cerebral white matter is characteristic of KSS [23]. The subcortical cerebral white matter is often abnormal. However, basal ganglia involvement like that which we observed in our patients has not been reported in any studies [24]. Cerebellar and/or cerebral atrophy were present in the older patients (cases 3, 4, 5 and 7), which is in accordance with the results reported by Chu et al. [20].

MRI abnormalities have been shown to increase in parallel with the neurologic progression of KSS [25], and we observed this pattern in patients 1, 2, 3 and 4. MRIs revealed progression in all of the cases with the exception of case 8. However, the interval between the acquisitions of the images was different in each case. Interestingly, all cases had ataxia except case 2. Some of the patients did not exhibit abnormalities in the cerebellum on the initial MRI but exhibited such abnormalities later. Interestingly, case 6 exhibited improvements in ataxia following folinic therapy but showed progression on MRI. In KSS, the correlations between cerebellar ataxia and MRI findings can be weak [20]. Furthermore, the effects of folinic acid therapy on brain are not completely understood, but probably they are wider than those related with neuroimaging changes, since brain is a target organ for folate deficiency. Otherwise, the progression of signal abnormalities coincides with neurologic deterioration. Therefore, follow-up MRIs might be useful for the assessment of the progression of this disease [26].

MRI signal changes do not necessarily indicate dysfunction. Nevertheless, DWI, which shows restrictions in diffusion and can detect acute ischemic lesions in stroke patients, might be capable of indicating acute energy deprivations. Abnormal DWI results were found in our patients. The pathophysiology of diffusion abnormalities that are associated with degenerative disorders such as mitochondrial diseases remain to be clarified [27]. The affected regions (particularly the brain stem) are more sensitive to energy deficiencies than are other brain areas and have been shown to be affected in patients with acute energy deprivation syndromes, such as Wernicke’s encephalopathy and Leigh syndrome. The pathologic features of the postmortem brains of patients with KSS include spongiform degenerations, necrosis and gliosis. Such changes are also present in other diseases such as Wilson disease and Creutzfeld-Jacob disease. These findings suggest that mechanisms other than cerebral folate deficiency could be involved in the neuroimaging abnormalities observed in KSS.

## Conclusions

Cerebral 5-MTHF deficiency was observed in all eight patients with KSS. Neuroimaging revealed lesion that have been reported to be characteristic of KSS. The majority of our patients exhibited clinical and radiological progression of the disease despite the restoration of normal CSF 5-MTHF values due to folinic treatment. Only one patient who was treated in the early stage of the disease exhibited neurological and radiological improvement following high-dose folinic acid therapy. Thus, the early initiation of high-dose folinic acid treatment seems advisable for KSS syndrome, at least to improve the neurological outcome.

## Additional files

Additional file 1:
**Case 1.** MRI axial T2W showed abnormal high signal in cerebellum (A), globus pallidus (B) and subcortical white matter (C). Additional file 2:
**Case 2.** Multi Vane T2-Weighted TSE (T2W-MV) disclosed hyperintensity in dorsal brain stem (A), globus pallidus atrophy (B), subcortical retraction and white matter hyperintensity (C). Additional file 3:
**Case 3.** DWI showed hyperintensity in brain stem (A), in left pallidum with restricted difussion (low ADC) (B) and in subcortical white matter with FLAIR (C). Additional file 4:
**Case 4.** Computed Tomography scan: White matter hypondensity in posterior fossa (A), basal ganglia (B) and subcortical white matter (C) was observed. Additional file 5:
**Case 6.** MRI axial T2W. Abnormal high signal in brainstem (A) and globus pallidus (B) was observed. The same lesions are shown in DWI (C,D).

## Abbreviations

KSS: Kearns-Sayre syndrome
mtDNA: Mitochondrial DNA
CSF: Cerebrospinal fluid
5-MTHF: 5-methyltetrahydrofolate
CNS: Central nervous system
MRIs: *Magnetic resonance imaging*
DWI: D*iffusion weighted* imaging
FLAIR: Fluid attenuated inversion recovery
CT: Computed tomography
RRF: Ragged red fibers
