# Supplementary figures and images for: Follow-up of folinic acid supplementation for patients with cerebral folate deficiency and Kearns-Sayre syndrome
Source: Orphanet J Rare Dis. 2014 Dec 24;9:217. doi: 10.1186/s13023-014-0217-2 (PMC4302586; doi:10.1186/s13023-014-0217-2)

## Slide 1
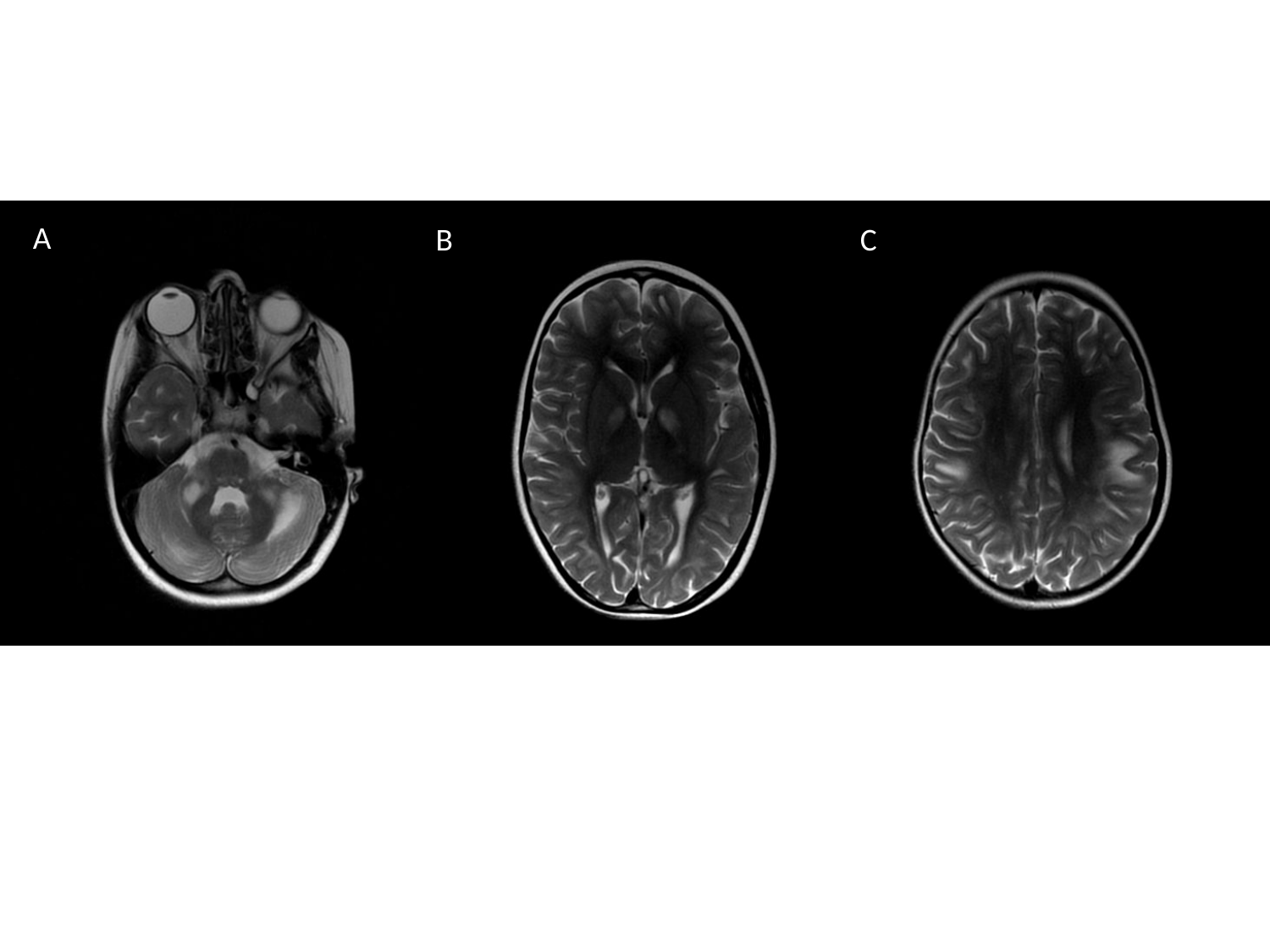

A
B
C

Supplement: Additional file 1: — Case 1. MRI axial T2W showed abnormal high signal in cerebellum (A), globus pallidus (B) and subcortical white matter (C). [file 13023_2014_217_MOESM1_ESM.pptx]

## Slide 1
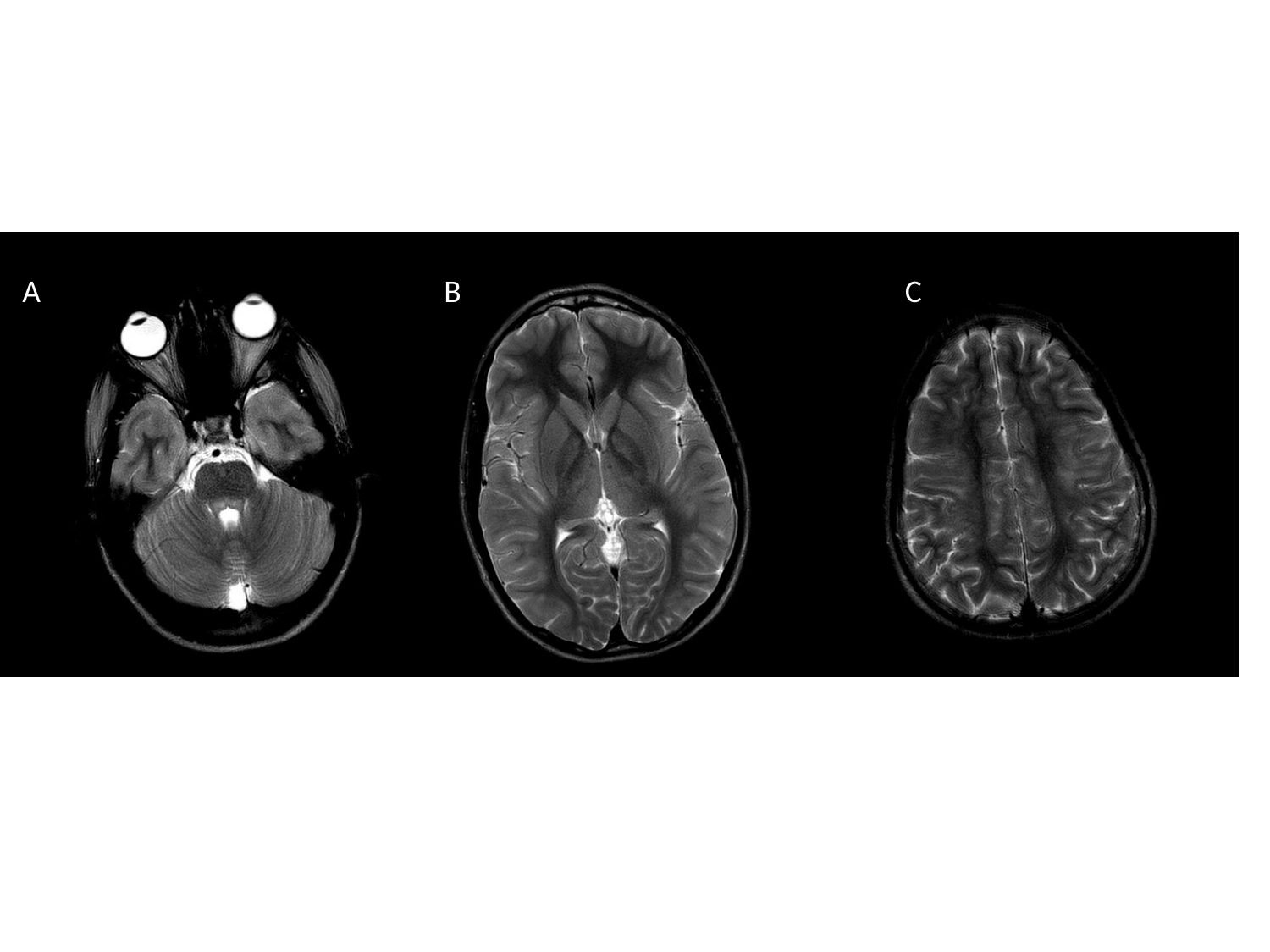

A
B
C

Supplement: Additional file 2: — Case 2. Multi Vane T2-Weighted TSE (T2W-MV) disclosed hyperintensity in dorsal brain stem (A), globus pallidus atrophy (B), subcortical retraction and white matter hyperintensity (C). [file 13023_2014_217_MOESM2_ESM.pptx]

## Slide 1
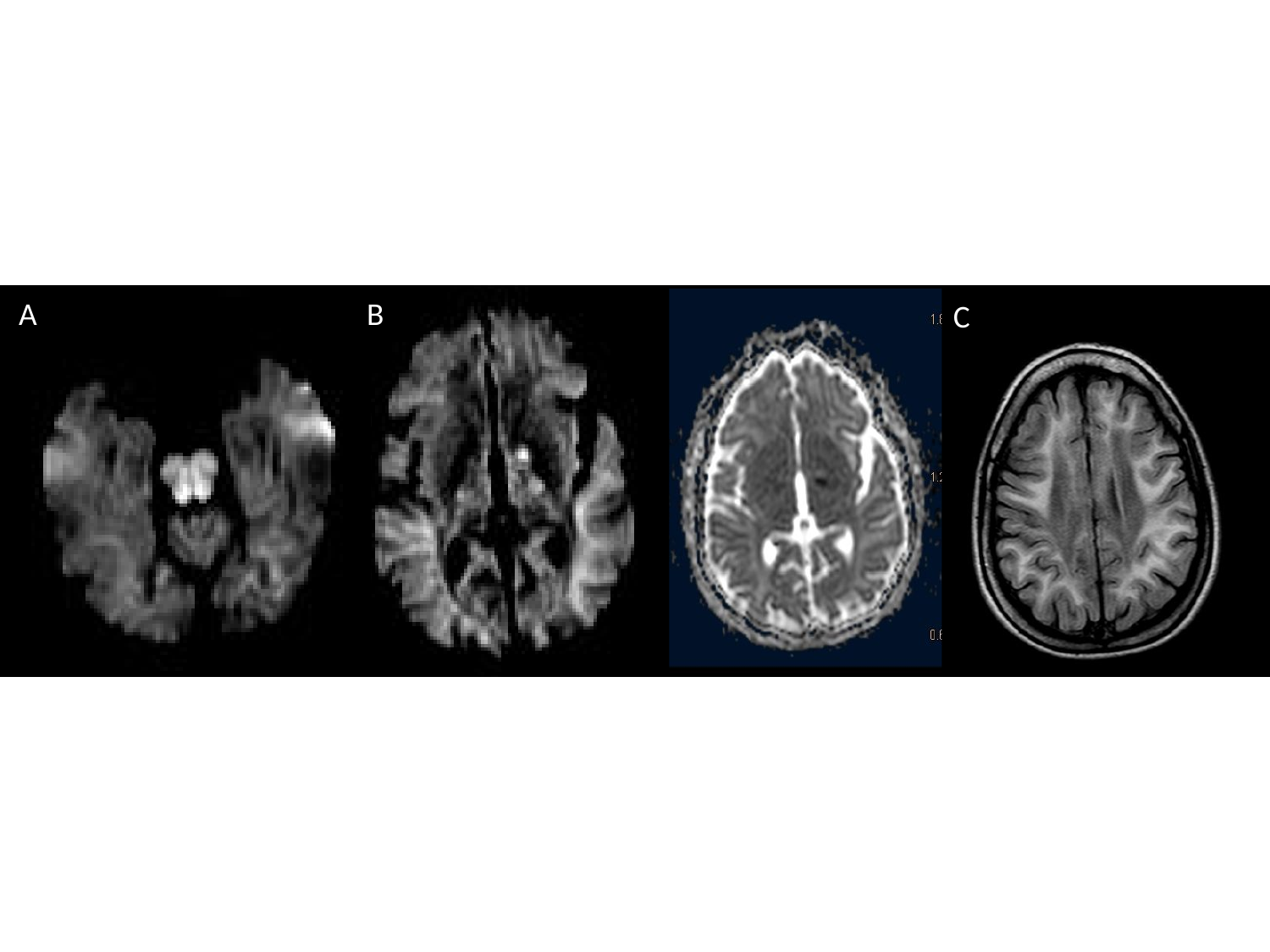

A
B
C

Supplement: Additional file 3: — Case 3. DWI showed hyperintensity in brain stem (A), in left pallidum with restricted difussion (low ADC) (B) and in subcortical white matter with FLAIR (C). [file 13023_2014_217_MOESM3_ESM.pptx]

## Slide 1
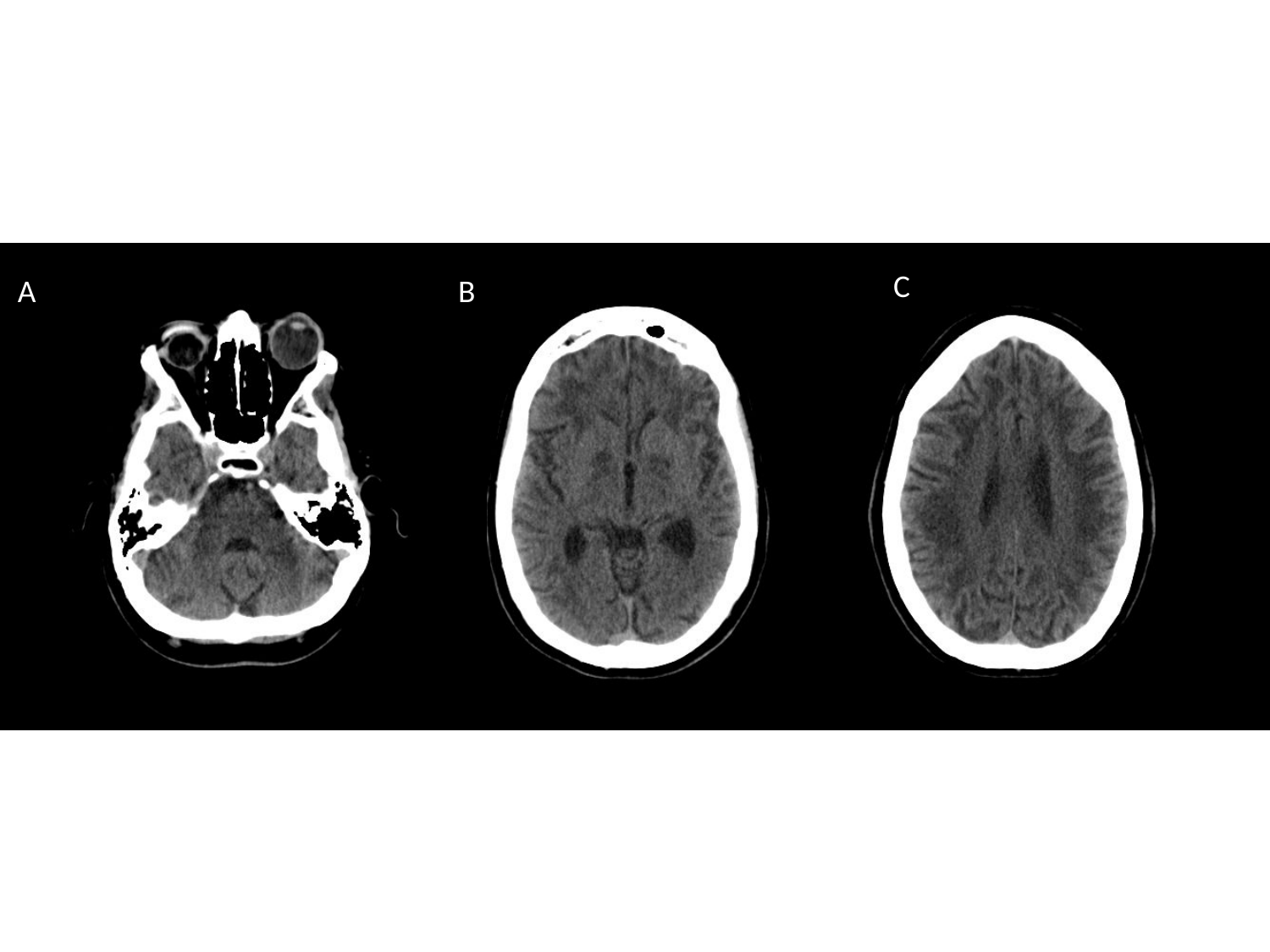

C
A
B

Supplement: Additional file 4: — Case 4. Computed Tomography scan: White matter hypondensity in posterior fossa (A), basal ganglia (B) and subcortical white matter (C) was observed. [file 13023_2014_217_MOESM4_ESM.pptx]

## Slide 1
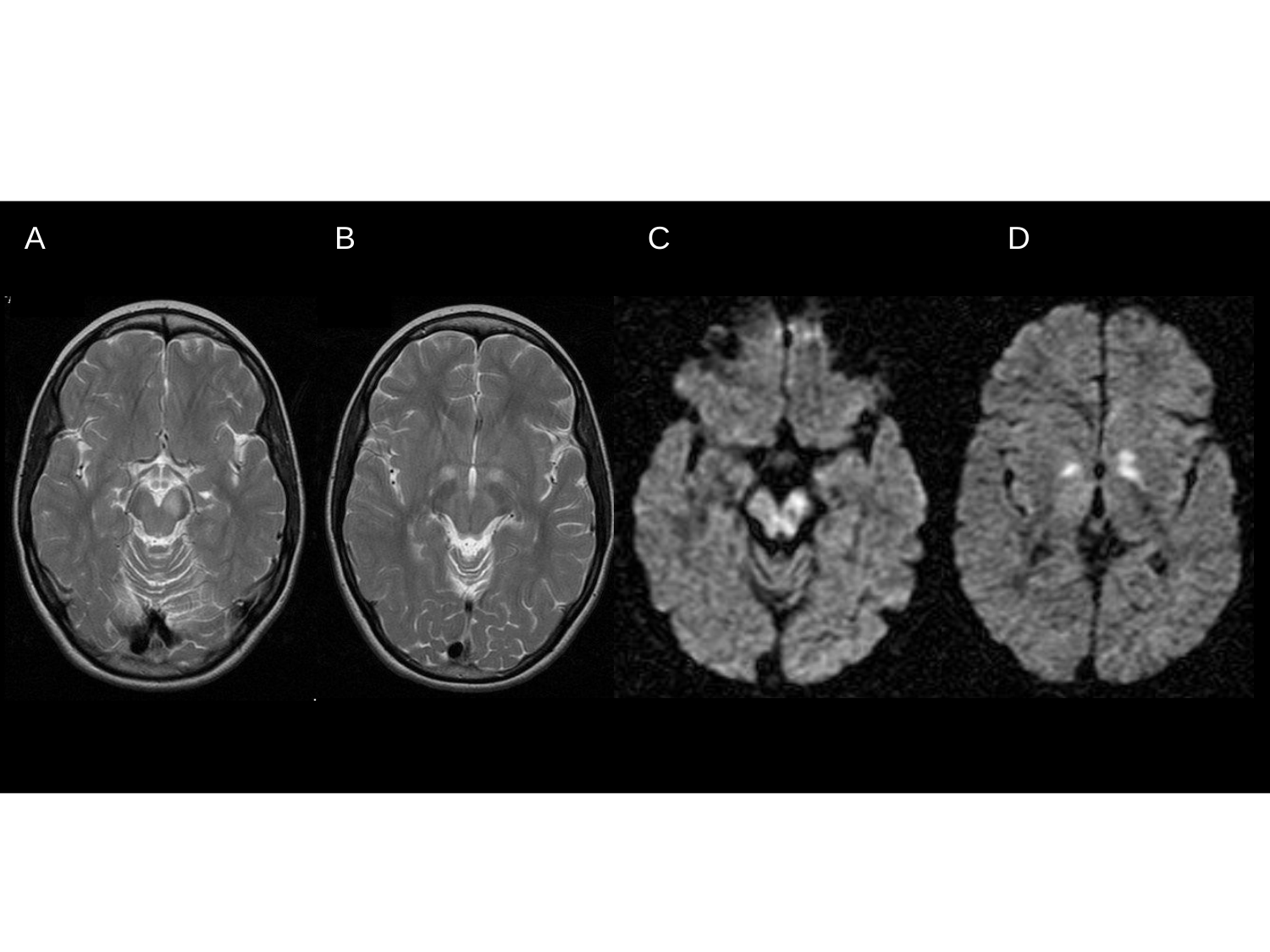

A
B
C
D

Supplement: Additional file 5: — Case 6. MRI axial T2W. Abnormal high signal in brainstem (A) and globus pallidus (B) was observed. The same lesions are shown in DWI (C,D). [file 13023_2014_217_MOESM5_ESM.pptx]
